# Pulmonary sequelae of pediatric patients after discharge for COVID‐19: An observational study

**DOI:** 10.1002/ppul.25239

**Published:** 2021-02-09

**Authors:** Che Zhang, Li Huang, Xiaoshi Tang, Yuxin Zhang, Xihui Zhou

**Affiliations:** ^1^ The First Affiliated Hospital of Xi'an Jiaotong University Xi'an Shaanxi China; ^2^ Affiliated Taihe Hospital of Hubei University of Medicine Shiyan Hubei China; ^3^ Prenatal Diagnostic Centre and Cord Blood Bank, Guangzhou Women and Children's Medical Centre, Guangzhou Medical University Guangzhou Guangdong China

## 1 INTRODUCTION

The coronavirus disease (COVID‐19) pandemic caused by the severe acute respiratory syndrome coronavirus 2 (SARS‐CoV‐2) has affected over 70,000,000 individuals globally; pediatric patients comprise 1%–5% of all diagnosed cases.^1^ Medical care recommendations are available for adults with COVID‐19 to facilitate recovery from pulmonary sequelae^2^; however, limited data are available regarding the pulmonary sequelae in pediatric patients discharged after COVID‐19 infection. In this observational study, we investigated the pulmonary manifestations in and clinical characteristics of 14 pediatric patients with COVID‐19, who underwent 30‐day follow‐up after hospital discharge.

## 2 CASE SERIES PRESENTATION

This study was approved by the Institutional Review Board of the Affiliated Taihe Hospital of Hubei University of Medicine (ethical approval no. 2020KY01). Pediatric patients with COVID‐19 were recruited from our previous study^3^; however, follow‐up data were reported only in this study. Written informed consent was obtained from the patients and their guardians before data were obtained from patients' medical charts. Statistical analysis was performed using SPSS software, version 20.0 (IBM). Statistical significance was set at *p* < .05.

**Figure 1:**
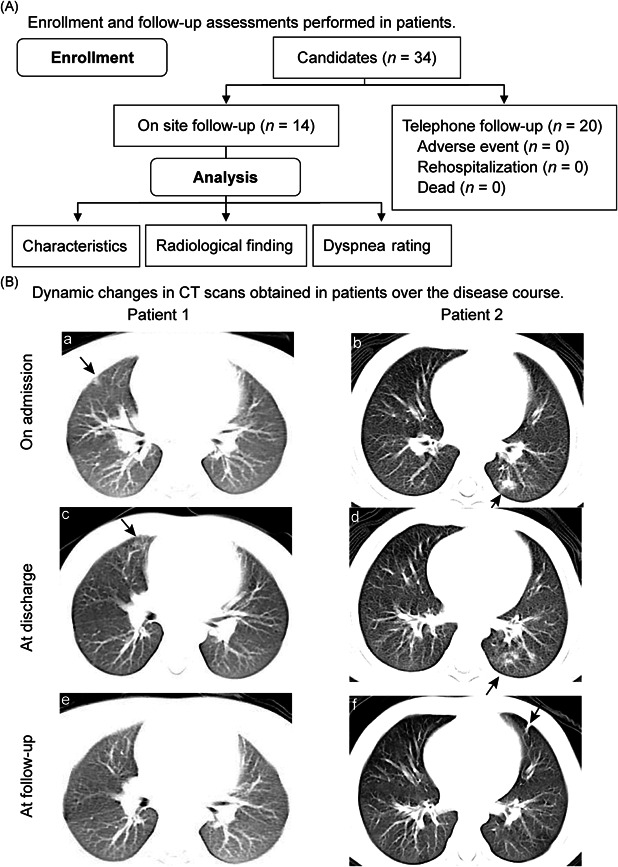
Clinical course of patients. A, Flow chart showing enrollment and follow‐up assessments performed in patients. Clinical data, including patient characteristics, radiological findings, and dyspnea score ratings were obtained from 14 patients during on‐site follow‐up visits. B, Images showing dynamic changes in CT scans obtained in patients over the disease course (a) Image obtained in Patient 1 on admission (February 20, 2020) showing a ground‐glass opacity in the middle lobe of the right lung. (b) Image obtained in Patient 2 on February 22, 2020, showing patchy high‐density shadows in the middle lobe of the left lung. (c) Image obtained in Patient 1 on February 26, 2020. This patient met the discharge criteria; however, a new lesion is observed in the middle lobe of the right lung. (d) Image obtained in Patient 2 at the time of discharge on March 12, 2020, showing significant improvement. (e) Image obtained in Patient 1 at the follow‐up visit on March 28, 2020, showing complete radiological resolution. (f) Image obtained in Patient 2 on April 10, 2020, showing a fibrous stripe close to the original lesion in the middle lobe of the left lung. Arrows showing the abnormal findings in the lung lobes. CT, computed tomography

The following were the inclusion criteria for this study: (1) diagnosis of COVID‐19,^4^ (2) discharge based on specific criteria and,^5^ (3) completion of computed tomography (CT) and other study‐specific evaluations 30 days (±7 days) post‐discharge. Among the 34 pediatric patients who were discharged from hospitals between March 15 and April 30, 2020, as reported in our previous study, 14 patients (boys:girls ratio 4:10) completed on‐site follow‐up, 30.1 (27.5–33.3) days post‐discharge (Table S1 in the Supporting Information), and 20 patients underwent telephone follow‐up and reported no adverse events (Figure 1A). No patient required rehospitalization.

Chest CT images were independently reviewed by two radiologists and were evaluated based on a score system that was described in the previous study^6^ (Table S2). The lesions were defined based on criteria utilized in a previous study.^7^ A complete radiological resolution was defined as the absence of potential infection‐induced abnormalities on chest radiography during follow‐up visits. CT scores observed during follow‐up were significantly improved compared with those observed at discharge (*p* < .05, Table S3). Pulmonary sequelae were observed in seven patients (7/14, 50%), 29.0 (26.0–33.0) days post‐discharge. These included spots or patches of opacities (3/14, 21%) and fibrosis (4/14, 29%, Table S3). A complete radiological resolution was observed in the other seven patients without any potential infection‐induced abnormalities (Figure 1B,e). Fibrosis manifested as parenchymal bands (Figure 1B,f) or a coarse reticular pattern in four patients. CT scores were higher in patients with pulmonary sequelae than in those with complete resolution at discharge and at follow‐up (*p* < .05, Table S3).

The dyspnea grades were evaluated during follow‐up using the Pediatric Respiratory Assessment Measure^8^ (Table S4) and the modified Medical Research Council dyspnea scales^9^ (Table S5) based on recommendations of the National Health Commission of the People's Republic of China.^2^ Oxygen therapy was not required in patients with pulmonary sequelae because rating scores showed only mild dyspnea in those affected. The scores recorded during follow‐up visits were lower than those observed at discharge (*p* < .05, Table S3), although no significant difference was observed between patients with and without pulmonary sequelae.

Tests for the SARS‐CoV‐2 RNA showed negative results at follow‐up in all patients. Hematological test results did not reveal any clinically significant abnormalities. The double‐antigen sandwich‐chemiluminescent magnetic microparticle immunoassay was used for the detection of SARS‐CoV‐2 antibodies, including immunoglobulin M (IgM) and immunoglobulin G (IgG). Tests for IgM and IgG showed negative results in all but one patient (1/14, 7%) with a positive IgG result. No infection was reported among close‐contact caregivers and patients' family members.

## 3 DISCUSSION

In this study, we investigated the pulmonary manifestations and clinical characteristics of 14 pediatric patients who underwent close follow‐up after hospital discharge. Radiological findings indicated that pulmonary sequelae occurred in a significant percentage (7/14, 50%) of patients, 30.1 (27.5–33.3) days post‐discharge, although no statistically significant difference was observed in dyspnea grades between patients with and without sequelae. The percentage was similar to that (47%) observed in adults with pulmonary sequelae, 21 days post‐discharge.^10^ Fibrosis was reported in 9% (14/149) of patients at the 21‐day follow‐up^10^ and in 44% (14/32) of patients at the 9‐day follow‐up.^11^ In our study fibrosis occurred in four (4/14, 29%) patients. The disparities in patient characteristics (e.g., age and general health condition), as well as different follow‐up periods across various studies, may have contributed to the differences in pulmonary sequelae observed between pediatric and adult patients. Notably, fibrosis was detected in pediatric patients, approximately 30 days post‐discharge. Fibrous stripes were detected at sites other than the original lesion in some patients (Figure 1B,f), which indicates the progressive nature of lesions, even after discharge. Therefore, pulmonary function monitoring is important in pediatric patients with pulmonary fibrosis.

Serological testing showed positive results for IgG in only one patient in contrast to results in adults, who usually show positive results for this test.^12^ The SARS‐CoV‐2 RNA test showed negative results in all patients in our study in contrast to the finding that 3%–9% of adults tend to show reactivation of SARS‐CoV‐2 post‐discharge.^13^ Moreover, SARS‐CoV‐2 infection was not reported among close‐contact caregivers and/or patients' family members. Further studies are warranted to investigate the dynamic changes in serum Ig levels, as well as the association between viral shedding and infectivity of pediatric patients post‐discharge.

The findings of this study contribute to expanding the understanding of the effects of COVID‐19 in pediatric patients discharged from the hospital. Specifically, pulmonary fibrosis requires close attention in this patient population.

Following are the limitations of this study: (a) Owing to the small sample size of this study, a detailed analysis was not possible to identify the risk factors for fibrosis. (b) A 30‐day follow‐up period was insufficient to accurately determine the long‐term pulmonary sequelae in children. Notwithstanding these limitations, our study results suggest that longer‐term monitoring of pulmonary function is necessary for further studies to gain a deeper understanding of the effects of COVID‐19 in pediatric patients.

## CONFLICT OF INTERESTS

The authors declare that there are no conflict of interests.

## AUTHOR CONTRIBUTIONS

**Che Zhang**: conceptualization (lead); data curation (lead); formal analysis (equal); investigation (lead); methodology (equal); resources (lead); software (equal); validation (equal); writing original draft (equal); writing review & editing (equal). **Li Huang**: conceptualization (equal); data curation (equal); formal analysis (lead); investigation (supporting); methodology (equal); resources (equal); software (lead); validation (equal); writing original draft (lead); writing review & editing (equal). **Xiaoshi Tang**: data curation (equal); investigation (equal); project administration (lead); resources (equal); validation (equal); writing review & editing (equal). **Yuxin Zhang**: data curation (supporting); investigation (equal); project administration (equal); resources (equal); validation (equal); writing review & editing (supporting). **Xihui Zhou**: conceptualization (equal); funding acquisition (lead); methodology (lead); resources (supporting); software (supporting); supervision (lead); validation (lead); writing review & editing (lead).

**KEYWORDS** COVID‐19, pulmonary fibrosis, pediatrics, respiratory infections, SARS‐CoV‐2

## Supporting information

Supporting information

## Data Availability

The data that supports the findings of this study are available in the supplementary material of this article.
