# Supplementary material for: Pulmonary sequelae of pediatric patients after discharge for COVID‐19: An observational study
Source: Pediatr Pulmonol. 2021 Feb 9;56(5):1266–9. doi: 10.1002/ppul.25239 (PMC8012994; doi:10.1002/ppul.25239)
Supplement: Supplementary file 1 — Supporting information [file PPUL-56-1266-s001.docx]

**Pulmonary sequelae of pediatric patients after discharge for COVID-19:**

**An observational study**

Che Zhang, Li Huang, Xiaoshi Tang, Yuxin Zhang, Xihui Zhou

**Supporting Document**

**E-table 1** Clinical characteristics of pediatric patients

**E-table 2** Lung CT scoring system for lesions in each lobe

**E-table 3** Radiological and dyspnea scores of pediatric patients

**E-table 4** The Pediatric Respiratory Assessment Measure Scale

**E-table 5** The modified Medical Research Council Scale

**E-table 1** Clinical characteristics of pediatric patients

| Clinical characteristics | All | Complete resolution | Pulmonary sequelae |
| --- | --- | --- | --- |
|  | (*n* = 14) | (*n* = 7) | (*n* = 7) |
| Gender |  |  |  |
| Male, *n* (%) | 4 (29) | 3 (43) | 1 (14) |
| Female, *n* (%) | 10 (71) | 4 (57) | 6 (86) |
| Age, median (IQR), m | 24.5 (9.0 – 54.8) | 22.0 (10.0 – 27.0) | 42.0 (6.0 – 98.0) |
| < 12 m | 4 (29) | 2 (29) | 2 (29) |
| 12 – 72m | 7 (50) | 5 (71) | 2 (29) |
| > 72 m | 3 (21) | 0 | 3 (42) |
| Initial symptom and sign |  |  |  |
| Fever, *n* (%) | 10 (71) | 6 (86) | 4 (57) |
| Cough, *n* (%) | 7 (50) | 2 (29) | 5 (71) |
| Expectoration, *n* (%) | 3 (21) | 0 | 3 (43) |
| Diarrhea, *n* (%) | 3 (21) | 2 (29) | 1 (14) |
| Vomiting, *n* (%) | 2 (14) | 0 | 2 (29) |
| Tachypnea, *n* (%) | 2 (14) | 0 | 2 (29) |
| Respiratory pathogen mix-infection, *n* (%) | 8 (57) | 5 (71) | 3 (43) |
| *Mycoplasma pneumoniae*, *n* (%) | 4 (29) | 1 (14) | 3 (43) |
| Influenza B virus, *n* (%) | 4 (29) | 2 (29) | 2 (29) |
| Influenza A virus, *n* (%) | 2 (14) | 1 (14) | 1 (14) |
| Parainfluenza virus, *n* (%) | 1 (7) | 0 | 1 (14) |
| Hospitalization, median (IQR), d | 8.0 (6.8 – 12.0) | 7.0 (5.0 – 12.0) | 9.0 (7.0 – 12.0) |
| Follow-up period, median (IQR), d | 30.1 (27.5 – 33.3) | 31.0 (28.0 – 34.0) | 29.0 (26.0 – 33.0) |

Abbreviations: IQR, interquartile range

**E-table 2** Lung CT scoring system for lesions in each lobe ^6^.

| Score | Involvement of lobe |
| --- | --- |
| 0 | None |
| 1 | < 25% |
| 2 | 26% – 49% |
| 3 | 50% – 75% |
| 4 | > 75% |

The scores for five lung lobes were added up to a total score.

**E-table 3** Radiological and dyspnea scores of pediatric patients (*n* = 14)

| Findings | On admission | At discharge | At follow-up |
| --- | --- | --- | --- |
| Features of CT images |  |  |  |
| Spots or patches of opacity, *n* (%) | 13 (93) | 8 (57) | 3 (21) |
| Ground-glass opacity, *n* (%) | 2 (14) | 1 (7) | 0 |
| Fibrosis, *n* (%) | 0 | 4 (29) | 4 (29) |
| CT scores |  |  |  |
| All patients, mean ± SE | 2.1 ± 0.2 | 1.6 ± 0.2 | 0.8 ± 0.3^a^ |
| Complete resolution (*n* = 7), mean ± SE | 1.9 ± 0.4 | 1.1 ± 0.3 | 0 |
| Pulmonary sequelae (*n* = 7), mean ± SE | 2.4 ± 0.2 | 2.1 ± 0.3^b^ | 1.6 ± 0.3^b^ |
| PRAM |  |  |  |
| All patients, mean ± SE | - | 0.8 ± 0.1 | 0.2 ± 0.1 ^c^ |
| Complete resolution (*n* = 7), mean ± SE | - | 0.6 ± 0.2 | 0 |
| Pulmonary sequelae (*n* = 7), mean ± SE | - | 1.0 ± 0.0 | 0.4 ± 0.2 |
| mMRC |  |  |  |
| All patients, mean ± SE | - | 0.7 ± 0.1 | 0.2 ± 0.1 ^c^ |
| Complete resolution (*n* = 7), mean ± SE | - | 0.4 ± 0.2 | 0 |
| Pulmonary sequelae (*n* = 7), mean ± SE | - | 1.0 ± 0.0 | 0.4 ± 0.2 |

^a^ *P* < 0.05, one-way ANOVA among CT scores at discharge and in follow-up.

^b^ *P* < 0.05, two-tail T-test between subgroups of complete resolution and pulmonary sequelae.

^c^ *P* < 0.05, two-tail T-test between patients at discharge and in follow-up.

Abbreviations: CT, computed tomography; mMRC, modified medical research council; PRAM, pediatric respiratory assessment measure; SE, standard error

**E-table 4** The Pediatric Respiratory Assessment Measure Scale ^8^.

| Score | 0 | 1 | 2 | 3 |
| --- | --- | --- | --- | --- |
| Suprasternal retractions | Absent | Absent | Present | Present |
| Scalene muscle contraction | Absent | Absent | Present | Present |
| Air entry | Normal | Decreased at bases | Widespread decrease | Absent/minimal |
| Wheezing | Absent | Expiratory only | Inspiratory and expiratory | Audible without stethoscope/silent chest with minimal air entry |
| O_2_-saturation | ≥ 95% | 92– 94% | < 92% | < 92% |

**E-table 5** The modified Medical Research Council Scale ^9^.

| Grade | Description of Breathlessness |
| --- | --- |
| 0 | I only get breathless with strenuous exercise |
| 1 | I get short of breath when hurrying on level ground or walking up a slight hill |
| 2 | On level ground, I walk slower than people of the same age because of breathlessness, or I have to stop for breath when walking at my own pace on the level |
| 3 | I stop for breath after walking about 100 yards or after a few minutes on level ground |
| 4 | I am too breathless to leave the house or I am breathless when dressing |

**References**

6. Bernheim A, Mei X, Huang M, Yang Y, Fayad ZA, Zhang N, Diao K, Lin B, Zhu X, Li K, et al. Chest CT findings in coronavirus disease-19 (COVID-19): Relationship to duration of infection. Radiology. 2020. <https://doi.org/10.1148/radiol.2020200463>. [published online ahead of print February 23, 2020].

8. Eggink H, Brand P, Reimink R, Bekhof J. Clinical scores for dyspnoea severity in children: A prospective validation study. PLoS One. 2016. 10.1371/journal.pone.0157724. [published online ahead of print 2016/07/08].

9. Curci C, Pisano F, Bonacci E, Camozzi DM, Ceravolo C, Bergonzi R, De Franceschi S, Moro P, Guarnieri R, Ferrillo M, et al. Early rehabilitation in post-acute COVID-19 patients: data from an Italian COVID-19 rehabilitation unit and proposal of a treatment protocol. A cross-sectional study. Eur J Phys Rehabil Med. 2020. <https://doi.org/10.23736/S1973-9087.20.06339-X>. [published online ahead of print 2020/07/16].
